# Utilization of a novel mobile application, “HBB Prompt”, to reduce Helping Babies Breathe skills decay

**DOI:** 10.1371/journal.pgph.0000705

**Published:** 2023-05-08

**Authors:** Natalie Hoi-Man Chan, Hasan S. Merali, Niraj Mistry, Ryan Kealey, Douglas M. Campbell, Shaun K. Morris, Santorino Data

**Affiliations:** 1 Division of Neonatology, Department of Pediatrics, University of California, San Francisco School of Medicine, San Francisco, California, United States of America; 2 Department of Pediatrics, Temerty Faculty of Medicine, University of Toronto, Toronto, Ontario, Canada; 3 Division of Pediatric Emergency Medicine, Department of Pediatrics, McMaster Children’s Hospital, McMaster University, Hamilton, Ontario, Canada; 4 Division of Paediatric Medicine, The Hospital for Sick Children, Toronto, Ontario, Canada; 5 Interactive Media Lab, University of Toronto, Toronto, Ontario, Canada; 6 Design Research, TD Bank Group, Toronto, Ontario, Canada; 7 Department of Paediatrics, St. Michael’s Hospital, Unity Health Toronto, Toronto, Ontario, Canada; 8 Division of Infectious Diseases, Centre for Global Child Health, and Child Health Evaluative Sciences, The Hospital for Sick Children, Toronto, Ontario, Canada; 9 Department of Pediatrics and Child Health, Mbarara University of Science and Technology, Uganda; 10 Consortium for Affordable Medical Technologies in Uganda (CAMTech Uganda), Mbarara, Uganda; PLOS: Public Library of Science, UNITED STATES

## Abstract

**Background:**

Helping Babies Breathe (HBB) is a newborn resuscitation training program designed to reduce neonatal mortality in low- and middle-income countries. However, skills decay after initial training is a significant barrier to sustained impact.

**Objective:**

To test whether a mobile app, HBB Prompt, developed with user-centred design, helps improve skills and knowledge retention after HBB training.

**Methods:**

HBB Prompt was created during Phase 1 of this study with input from HBB facilitators and providers from Southwestern Uganda recruited from a national HBB provider registry. During Phase 2, healthcare workers (HCWs) in two community hospitals received HBB training. One hospital was randomly assigned as the intervention hospital, where trained HCWs had access to HBB Prompt, and the other served as control without HBB Prompt (NCT03577054). Participants were evaluated using the HBB 2.0 knowledge check and Objective Structured Clinical Exam, version B (OSCE B) immediately before and after training, and 6 months post-training. The primary outcome was difference in OSCE B scores immediately after training and 6 months post-training.

**Results:**

Twenty-nine HCWs were trained in HBB (17 in intervention, 12 in control). At 6 months, 10 HCW were evaluated in intervention and 7 in control. In intervention and control respectively, the median OSCE B scores were: 7 vs. 9 immediately before training, 17 vs. 21 immediately after training, and 12 vs. 13 at 6 months after training. Six months after training, the median difference in OSCE B scores was -3 (IQR -5 to -1) in intervention and -8 (IQR -11 to -6) in control (p = 0.02).

**Conclusion:**

HBB Prompt, a mobile app created by user-centred design, improved retention of HBB skills at 6 months. However, skills decay remained high 6 months after training. Continued adaptation of HBB Prompt may further improve maintenance of HBB skills.

## Introduction

Neonatal mortality continues to be a critical problem worldwide. The first 28 days of a child’s life is the most vulnerable period for survival and, in 2020, 2.4 million newborns died in this period [1]. Sub-Saharan Africa continues to have a very high neonatal mortality rate (NMR), with 27 deaths per 1000 live births, including Uganda which is estimated to have an NMR of 19 [1]. If we are to achieve the Sustainable Development Goal (SDG) of reducing the NMR to 12 by 2030, then significant advancement in providing quality healthcare by skilled health workers is crucial [2].

Helping Babies Breathe (HBB) is an evidence-based newborn resuscitation training program designed to be used in low-resource settings [3]. Utilising the principles of adult learning, HBB training sessions incorporate small group discussion, peer-to-peer facilitation, simulation, and debriefing. Sessions are run with one instructor and six participants split up into groups of two. Each pair uses a NeoNatalie (Laerdal Global Health) simulator, a low-cost mannequin that can demonstrate chest wall excursion and an umbilical pulse [4]. Since 2010, HBB has reached an estimated 850,000 providers in 158 countries [5]. In large-scale studies, HBB has been shown to decrease early neonatal mortality and fresh stillbirths in low- and middle-income countries (LMICs) [6–8]. One of the primary challenges for HBB, as with any educational program however, is the knowledge and skills drop-off that occurs over time [9–13]. Frequent practice both with self and peer-to-peer learning [11, 14, 15], as well as expert feedback have been shown to be helpful methods to sustain competencies [16]. In a recent qualitative study examining 10 years of HBB facilitator data, one of the themes that emerged as critical to the program was “frequent and sustained hands-on practice during and after the course [5].”

One method to facilitate low-dose high frequency (LDHF) practice is the use of mobile applications [17–20]. Mobile applications lend themselves well in assisting HCWs, given the: ubiquity of smartphone availability in LMICs [21, 22], the cost savings of using mobile health technologies (mHealth) [23], and, as the global COVID-19 pandemic has shown, the importance of being able to access this information outside of a traditional classroom-based approach [24]. In the decade since HBB was launched, several digital support tools have been created to assist facilitators and providers with data collection, education and training, clinical decision support, and quality improvement [25]. The applications that assist with education and training provide digital information [26–29], questions [27–29], case-scenarios for practice [29], and, more recently, virtual reality (VR) scenarios that can be used with low-cost VR headsets [25, 30]. It is within this context that a novel mobile application, HBB Prompt, was created in 2019 [31]. This mobile application was created using user-centered design (UCD) [32] and has four parts, including: Training mode, to demonstrate with videos the steps of the HBB algorithm; Simulation mode, to facilitate individual and peer-to-peer practice of resuscitation scenarios; Quizzes/Knowledge check, to refresh knowledge about HBB; and a Scoreboard/Dashboard that summarises user performance and app usage, to increase user motivation and accountability for maintaining skills.

The purpose of this study is to report on the results of our comparative analysis, which took place at two hospitals in Southwestern Uganda.

## Methods

### Study overview

This was a comparative study at two hospitals in Southwestern Uganda, in collaboration with the Mbarara University of Science and Technology (MUST). Details of the protocol can be found in the study’s published protocol paper [31]. The study (Clinicaltrials.gov: NCT03577054) consisted of two phases. Phase 1 was the development phase where HBB Prompt was created via an iterative approach utilizing UCD and focus group discussions (FGD) [32]. Phase 2 is the assessment phase where we collected data at the two hospitals—one that was exposed to the app, and the other that was not. This paper focuses on Phase 2 of the study. One significant change from our protocol is that we were only able to follow the defined outcomes to six months, rather than one year. This was due to the COVID-19 pandemic that occurred in the second half of our data collection period, which restricted in-person assessments, gatherings for group practice, and required redeployment for some of the HCWs in the study.

### Objectives

At the outset of the study, we had three main objectives. The first was completed in Phase 1 of the study and that was to develop HBB Prompt through UCD and input from frontline birth attendants in Uganda. The remaining two objectives for this study, modified for the follow-up timeframe, were:

1. Train a minimum of 20 health workers from two district hospital in Southwestern Uganda in HBB.
2. Determine the impact of HBB Prompt on HBB skills retention of providers in the intervention site compared with providers in the control site as measured by the HBB version 2.0 OSCE B test scores.

### Participants and hospital intervention

Participants for Phase 2 of this study included frontline birth attendants from two district level hospitals in Southwestern Uganda that have HBB providers but not trainers. Written consent to participate was obtained both from the medical superintendent at each hospital and the individual birth attendants. Using a random sequence generator Kitagata Hospital was randomly assigned to be the intervention hospital and Itojo Hospital was assigned to be the control hospital.

### HBB Prompt

#### User-centred design of HBB Prompt

HBB Prompt was designed in Phase 1 of this study with a participatory UCD approach. HBB trainers and providers were invited to participate in an iterative process to help design a tool to improve HBB learning and skills maintenance. Participants provided input regarding the process of learning and maintenance of HBB skills and knowledge, which informed the development of the key components of HBB Prompt. Subsequently, participants interacted with a prototype version of HBB Prompt (v1) and gave feedback through FGDs to further optimize the functionality, content, flow, and usability of the app. For example, peer-to-peer learning and timely feedback were identified as crucial aspects of HBB skills maintenance; therefore, HBB Prompt was designed to have Simulation mode to help facilitate real-time simulation of applying HBB skills to newborn care. Within Simulation mode, rater mode shows users an HBB checklist that can be used to provide feedback serving as a peer-to-peer learning tool. Further details can be found in our publication outlining the details of HBB Prompt [32].

#### HBB Prompt app features

**Fig 1:**
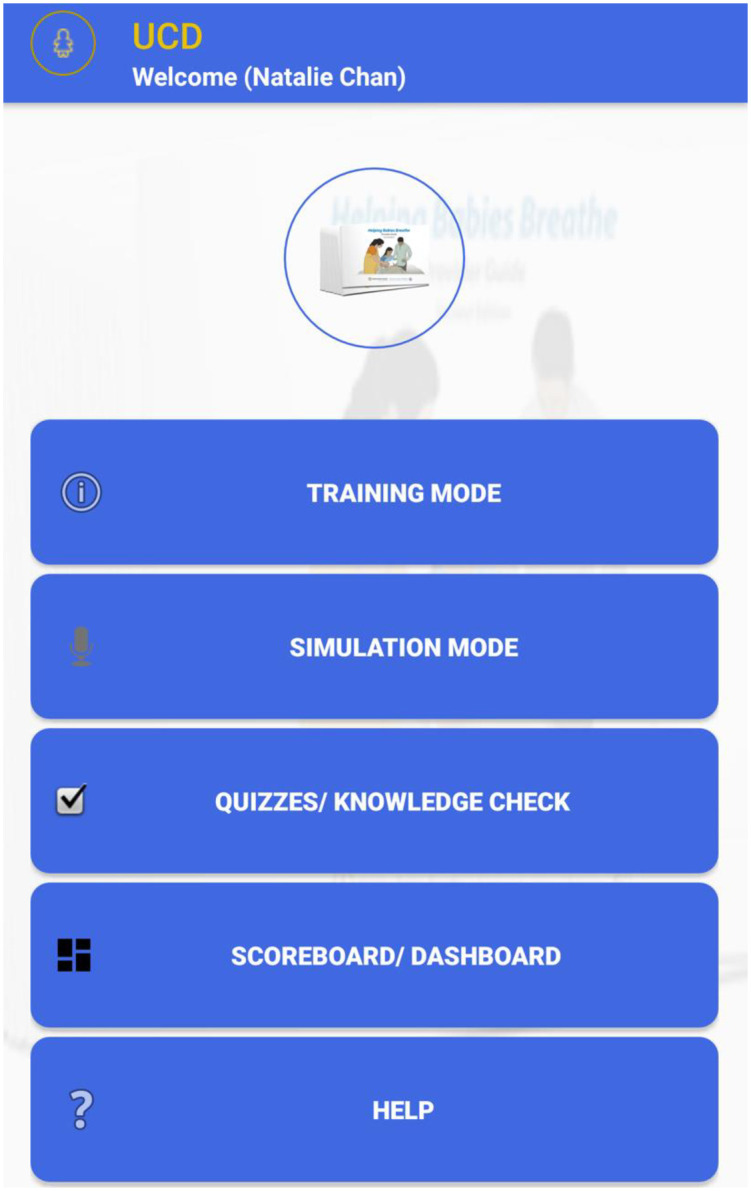
Screenshot from HBB Prompt app. Main menu screen for HBB Prompt showing the four main app features: Training mode, Simulation Mode, Quizzes/Knowledge check and Scoreboard/Dashboard.

HBB Prompt v2 was available to participants in the intervention hospital for the duration of the study. The key components of the app included Training mode, Simulation mode, Quizzes/Knowledge check, and a Scoreboard/Dashboard (Fig 1). Training mode consisted of a series of videos to demonstrate various steps in the HBB Action Plan and how to reprocess equipment. Simulation mode assisted providers in going through HBB practice scenarios in real time either alone or with a partner. Simulation mode was structured to transition in three phases: preparation, scenario execution and review with the checklist. In the preparation phase, users are provided the scenario stem and time to assemble all equipment and supplies for the scenario. During the scenario, the app displays the state of the baby using audio cues and associated graphics from the HBB Action Plan. Users are expected to follow the HBB Action Plan to appropriately manage the baby as the scenario progresses. At the end of the scenario phase, a performance checklist for that scenario appears on the screen to aid personal reflection on performance or peer feedback. The Quizzes/Knowledge check section provided practice questions to users with feedback to assist with retention of key pieces of information. Finally, the Scoreboard/Dashboard feature allowed users to see personal performance as well as comparison with other users.

### Phase 2

Following recruitment, each HCW underwent standard HBB training in-person with a Master Trainer. Separate sessions were held at each of the two hospitals. A skills corner was set-up in each hospital that had a NeoNatalie simulator and resuscitation equipment so that participants could practise their skills. Participants in both hospitals were asked to practise their skills at least once per shift and record practice sessions in a logbook. In addition to training, the intervention hospital was provided with HBB Prompt on one designated Android tablet computer for the duration of the study. Intervention group participants were asked to use HBB Prompt as part of their regular skills practice. The control group did not have access to HBB Prompt and it was not made publicly available for download. Data analytics were stored on the tablet and subsequently downloaded to the HBB Prompt server at the end of the study. Phase 2 took place from April to October 2019.

### Measurement of outcomes

During initial training, and at the 6-month follow-up, each participant completed a knowledge check, and OSCE B from the HBB program. At the end of the study, participants completed a questionnaire regarding their perception of HBB Prompt and had exit FGDs. See S1 and S2 Texts. The following outcomes were measured:

Primary Outcome

1. Comparison of OSCE B scores in intervention versus control group at 6 months after training

Secondary Outcomes

1. Comparison of knowledge assessments in intervention and control group before initial training and after 6 months.
2. App analytics in the intervention group to assess pattern of usage, frequency of app use, and scores in quizzes.
3. Exit FGDs at both sites to understand the impact of HBB Prompt and HBB training.

### Data processing and analysis

Stata Corporation, Version 13.1 was used to perform statistical analyses. Descriptive statistics were used to compare baseline characteristics of participants in the intervention and control groups. OSCE B and knowledge check scores were compared using Wilcoxon ranked-sum analysis. In the intervention arm, app analytic data on practice frequency were compared with logbook data using the Kappa statistic. For the FGDs, audio recordings were transcribed and then qualitatively analyzed to identify major themes. For the intervention group, multivariate linear regression models were used to assess variables that may confound the primary outcome, including: frequency of practice and frequency of app usage. For the purposes of this study, only simulation practice was recorded and analyzed, and not any clinical practice outside of the study protocol.

### Ethics approval

This study received approval from the Research Ethics Board at The Hospital for Sick Children (Sickkids) (REB no. 1000059992) and the Research Ethics Committee at MUST (REC no. 16/09-17).

## Results

**Fig 2:**
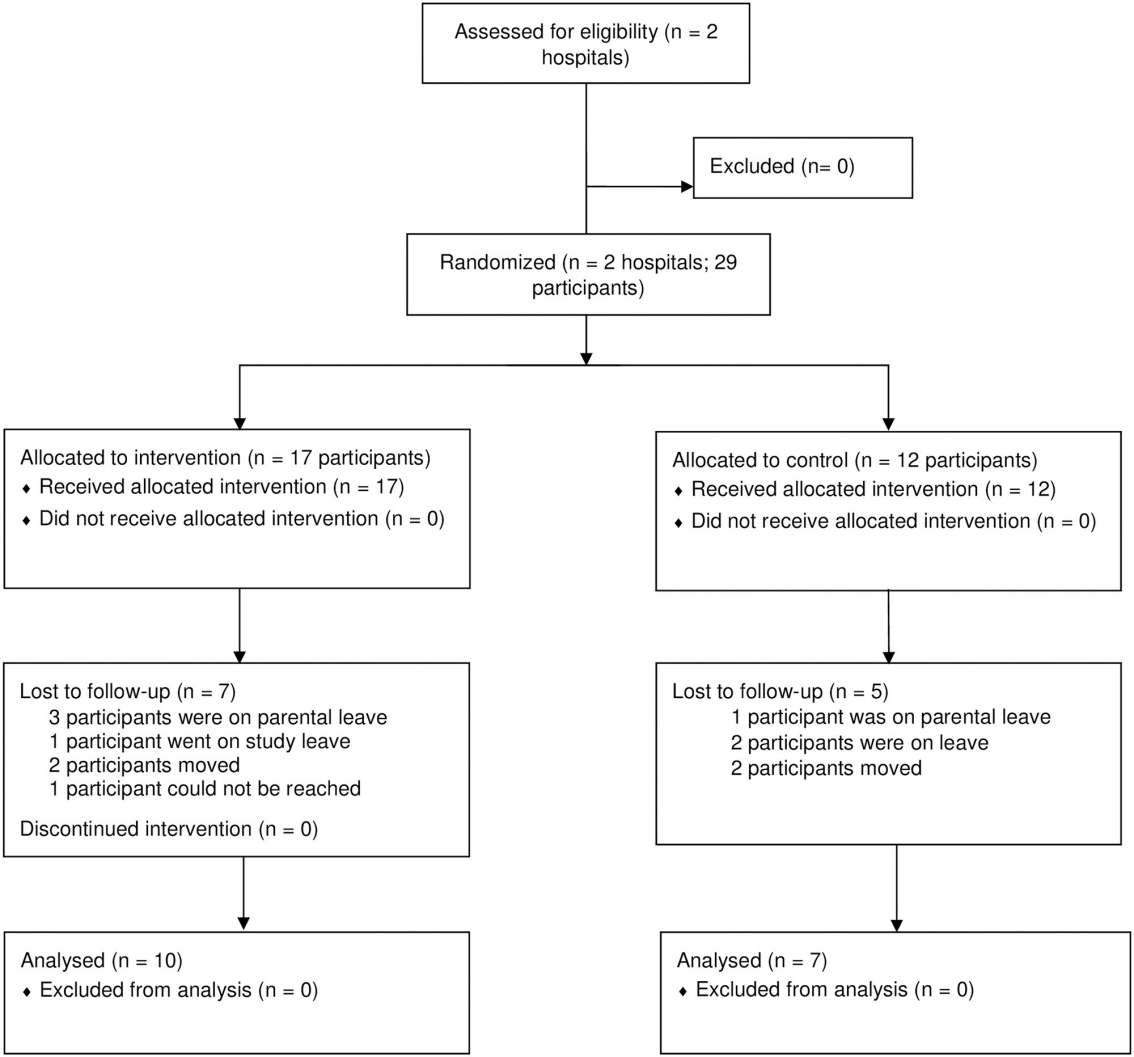
Participant flow diagram.

In total, 29 HCWs were recruited for the study: 17 in the intervention cohort and 12 in the control cohort (Fig 2). Participant demographic and clinical characteristics were similar across the two sites (Table 1).

**Table 1:** Baseline characteristics of providers in each hospital.

	Intervention (n = 17)	Control (n = 12)
Provider Type	Midwife = 14	Midwife = 11
	Enrolled Midwife = 1	Senior Nursing Officer = 1
	Anesthetic Officer = 1	
	Senior Nursing Officer = 1	
Gender	Female = 17	Female = 12
Years of experience as healthcare provider	Average = 11.4 years	Average = 9.5 years
	Median = 10 years	Median = 9 years
	Range = 2–28 years	Range = 3–18 years
	0–5 years = 5	0–5 years = 3
	6–10 years = 6	6–10 years = 4
	11–15 years = 0	11–15 years = 3
	15+ years = 6	15+ years = 2
Deliveries attended in the last month (at the time of training)	Average = 17.1	Average = 14.4
	Median = 20	Median = 15
	0–10 = 6	0–10 = 5
	11–20 = 4	11–20 = 4
	21–30 = 4	21–30 = 3
	30+ = 3	30+ = 0
Training courses previously attended		
Helping Babies Breathe	3	5
Helping Mothers Survive	1	3
BEMONC^1^	2	1
CEMONC^1^	2	0
ECEB^1^	1	0

^1^Basic Emergency Maternal and Newborn Care (BEMONC), Comprehensive Emergency Maternal and Newborn Care (CEMONC), Essential Care for Every Baby (ECEB)

**Fig 3:**
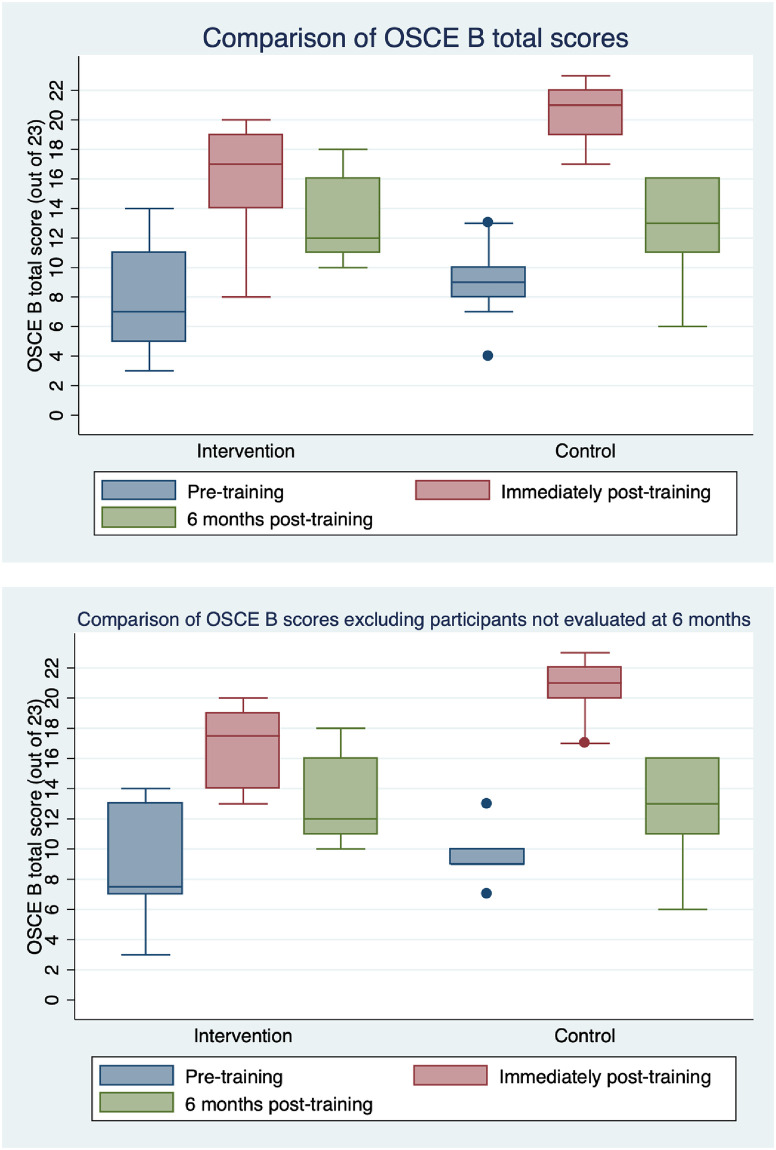
Comparison of distribution of OSCE B scores immediately before and after training, and six months after training. (A) Both groups had significantly improved scores immediately after training (*p* = 0.0003 in intervention, *p* = 0.002 in control). (B) When those lost-to follow-up were excluded, both groups still had significantly improved scores immediately after training. Within each group, there was also a significant decrease in scores six months post-training when compared with immediately after training (*p* = 0.006 in intervention, *p* = 0.02 in control). The intervention group showed less skills decay after six months, as indicated by smaller change in median difference of OSCE B scores (*p* = 0.02).

**Fig 4:**
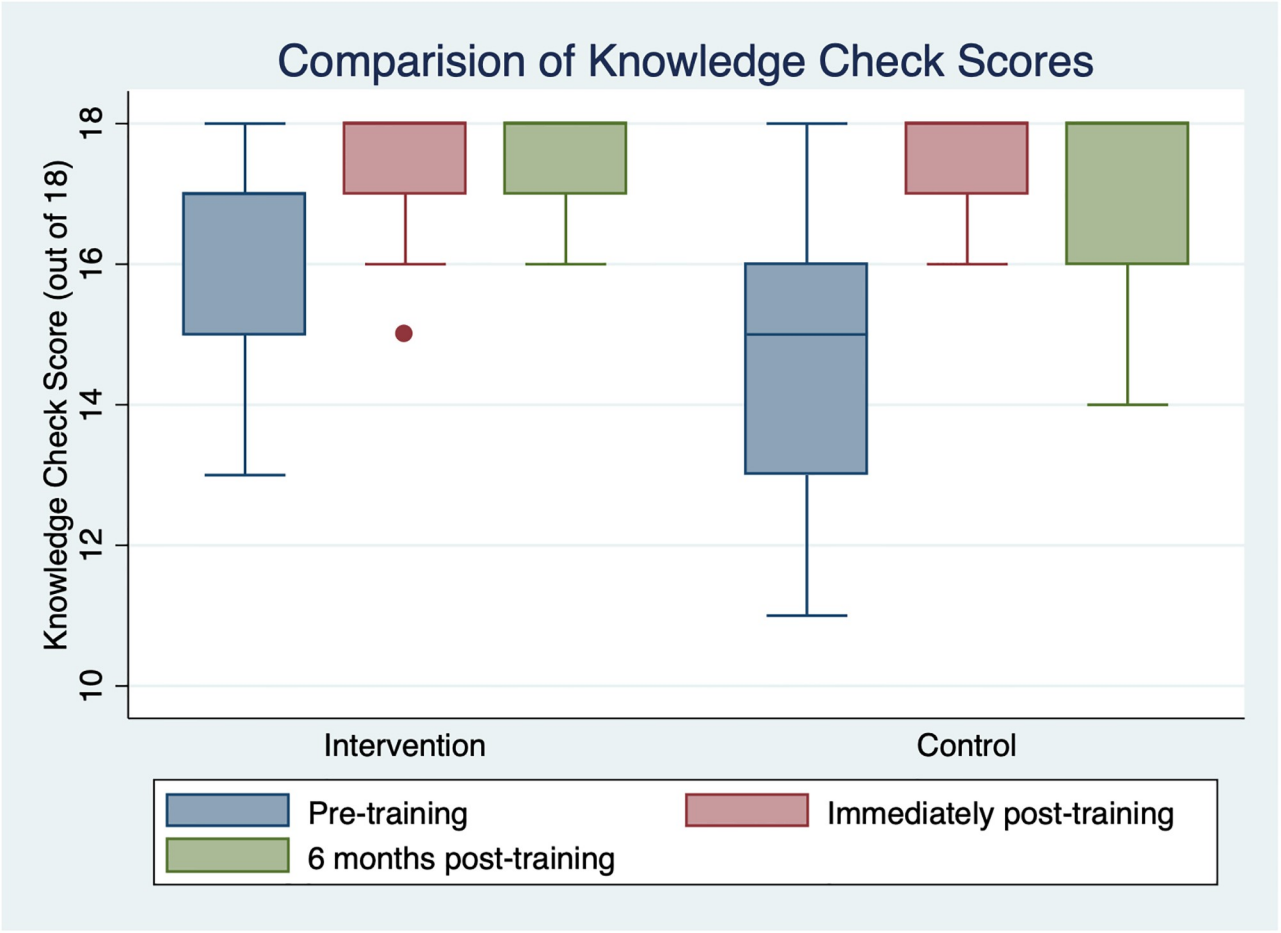
Comparison of distribution of knowledge check scores immediately before and after training, and six months after training. Both groups showed improvement in knowledge after training (*p* = 0.004 in intervention, *p* = 0.003 in control). In contrast to skills retention, knowledge retention remained high in both groups with no significant decay (*p* = 1 in intervention, *p* = 0.66 in control).

Six months after HBB training, 10 HCWs were evaluated in the intervention group and 7 in the control arm. The OSCE B has a total score of 23, and the knowledge check has a maximum score of 18. When examining only participants who were evaluated six months after training, there was a statistically significant difference in the OSCE B scores with a median difference of -3 (IQR -5 to -1) in the intervention group and -8 (IQR -11 to -6) in the control group (p = 0.02). The median difference in knowledge check scores was 0 (IQR 0 to 0) in the intervention group and 0 (IQR -1 to 1) in the control group (p = 0.68). Fig 3A and Table 2 illustrate the distribution of OSCE B scores at different time points for every participant evaluated at all time points including those who were lost-to follow-up at six months. Fig 3B shows only the participants who were evaluated at six months. Fig 4 and Table 3 show the distribution of knowledge check scores. 17 out of 23 is considered a passing score and only 2 participants (both in the intervention arm) had passing scores.

**Table 2:** Comparison of OSCE B scores pre-training, immediately post-training and 6 months post-training.

	Intervention	Control	p-value
**All participants**	n = 17	n = 12	
**Pre-training** Median (IQR)	7 (IQR 5 to 11)	9 (IQR 7.5 to 10)	p = 0.36
**Immediately post-training** Median (IQR)	17 (IQR 14 to 19)	21 (IQR 18.5 to 22)	*p = 0*.*0009*
**Only participants evaluated at 6 months**	n = 10	n = 7	
**Pre-training** Median (IQR)	7.5 (IQR 7 to 13)	9 (IQR 9 to 10)	p = 0.46
**Immediately post-training** Median (IQR)	17.5 (IQR 14 to 19)	21 (IQR 20 to 22)	*p = 0*.*0045*
**6 months post-training** Median (IQR)	12 (IQR 11 to 16)	13 (IQR 11 to 16)	p = 0.77
**Median difference 6 months vs. immediately post-training** Median (IQR)	-3 (IQR -5 to -1)	-8 (IQR -11 to -6)	*p = 0*.*02*

**Table 3:** Comparison of knowledge check scores pre-training, immediately post-training and 6 months post-training.

	Intervention	Control	p-value
**All participants**	n = 17	n = 12	
**Pre-training** Median (IQR)	17 (IQR 15 to 17)	15 (IQR 13 to 16)	p = 0.09
**Immediately post-training** Median (IQR)	18 (IQR 17 to 18)	18 (IQR 17 to 18)	p = 0.84
**Only participants evaluated at 6 months**	n = 10	n = 7	
**Pre-training** Median (IQR)	16.5 (IQR 15 to 17)	16 (IQR 13 to 16)	p = 0.42
**Immediately post-training** Median (IQR)	18 (IQR 17 to 18)	18 (IQR 17 to 18)	p = 0.87
**6 months post-training** Median (IQR)	18 (IQR 17 to 18)	18 (IQR16 to 18)	p = 0.66
**Median difference 6 months vs. immediately post-training** Median (IQR)	0 (IQR 0 to 0)	0 (IQR -1 to 1)	p = 0.68

Lost-to follow-up was similarly high in both groups, 7 of 17 (41%) participants in intervention and 5 of 12 (42%). Reasons for lost-to follow-up were also similar, including leaves of absences and moving away from the district (Fig 2). For participants evaluated at six months, median deliveries per month at the start of training and years of experience did not significantly differ between the intervention and control groups (Table 4). Within each group, participants who were evaluated compared with those who were lost to follow-up did not differ in their years of experience as a healthcare provider. In the intervention group, participants who were evaluated attended less deliveries per month compared to those who were lost-to follow-up, 15 (IQR 0 to 20) vs. 30 (IQR 20 to 32), *p* = 0.03.

**Table 4:** Differences in characteristics of participants evaluated 6 months after HBB training versus those lost-to follow-up.

	Evaluated	Lost-to follow-up	P-value
**Intervention**	**n = 10**	**n = 7**	
Median deliveries per month	15 (IQR 0 to 20)	30 (IQR 20 to 32)	p = 0.03
Median years of experience as healthcare provider	8.5 (IQR 5 to 16)	10 (IQR 5 to 18)	p = 0.92
**Control**	**n = 7**	**n = 5**	
Median deliveries per month	15 (IQR 6 to 20)	15 (IQR 10 to 26)	p = 0.57
Median years of experience as healthcare provider	10 (IQR 6 to 12)	8 (IQR 3.5 to 15)	p = 0.94

Average delivery volume per month was 350 in the intervention site and 200 in the control site. Three-month running average rates of infants with asphyxia and fresh stillbirths are shown in Table 5. Birth asphyxia was defined as a failure to initiate or sustain spontaneous breathing at birth [33]. Compared to the baseline rate in the three months prior to HBB training, the rate of infants with asphyxia decreased significantly in the intervention site six to nine months after HBB training (*p* = 0.0004). When compared to baseline rates at each site, there was a downward trend in fresh stillbirth rates in the intervention site that was not statistically significant (*p* = 0.37) while there was an upward trend in the control site (*p* = 0.43).

**Table 5:** Rates of fresh stillbirth and infants with asphyxia in intervention and control sites during pre- and post-HBB training periods.

	3 months before HBB training (pre intervention period)	1–3 months after HBB training	4–6 months after HBB training	6–9 months after HBB training (post intervention period)	Chi-square p-value comparison of pre vs. post intervention period
**Fresh Stillbirths per 1000 total births**					
Intervention	7.4	7.0	4.1	4.4	p = 0.37
Control	9.5	17.1	14.0	15.2	p = 0.43
**Infants with asphyxia per 1000 live births**					
Intervention	60.5	26.2	33.2	32.9	*p = 0*.*0004*
Control	54.6	48.9	21.9	39.4	p = 0.19

### App usage in intervention group

**Fig 5:**
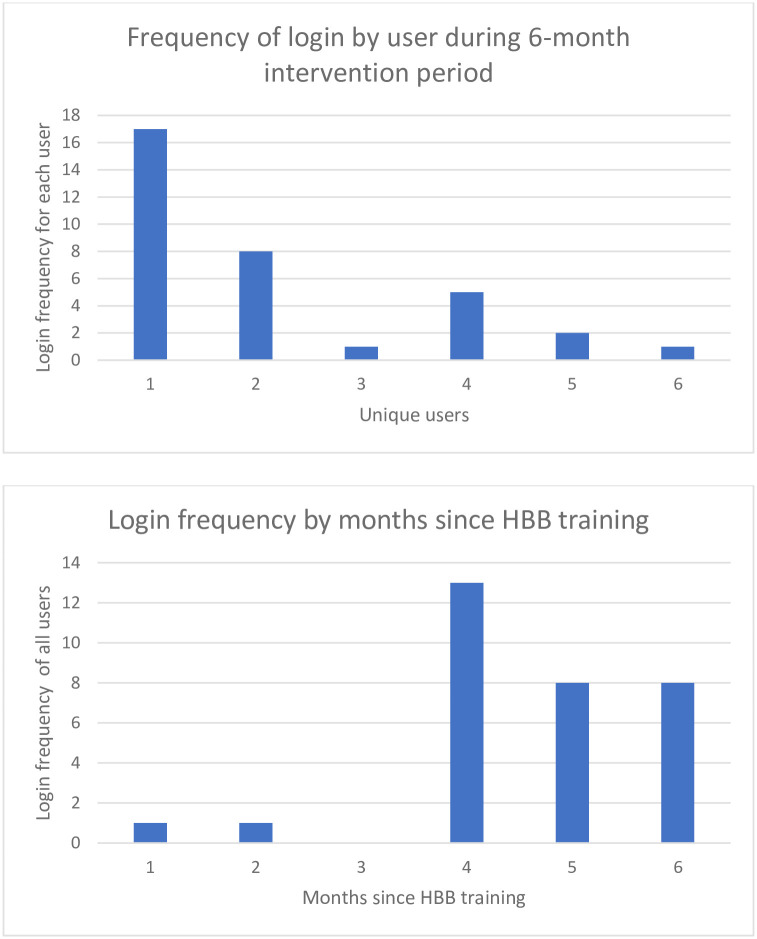
HBB Prompt usage over the duration of the intervention period. (A) Usage by login frequency per user and (B) usage over time, by months since HBB training.

**Fig 6:**
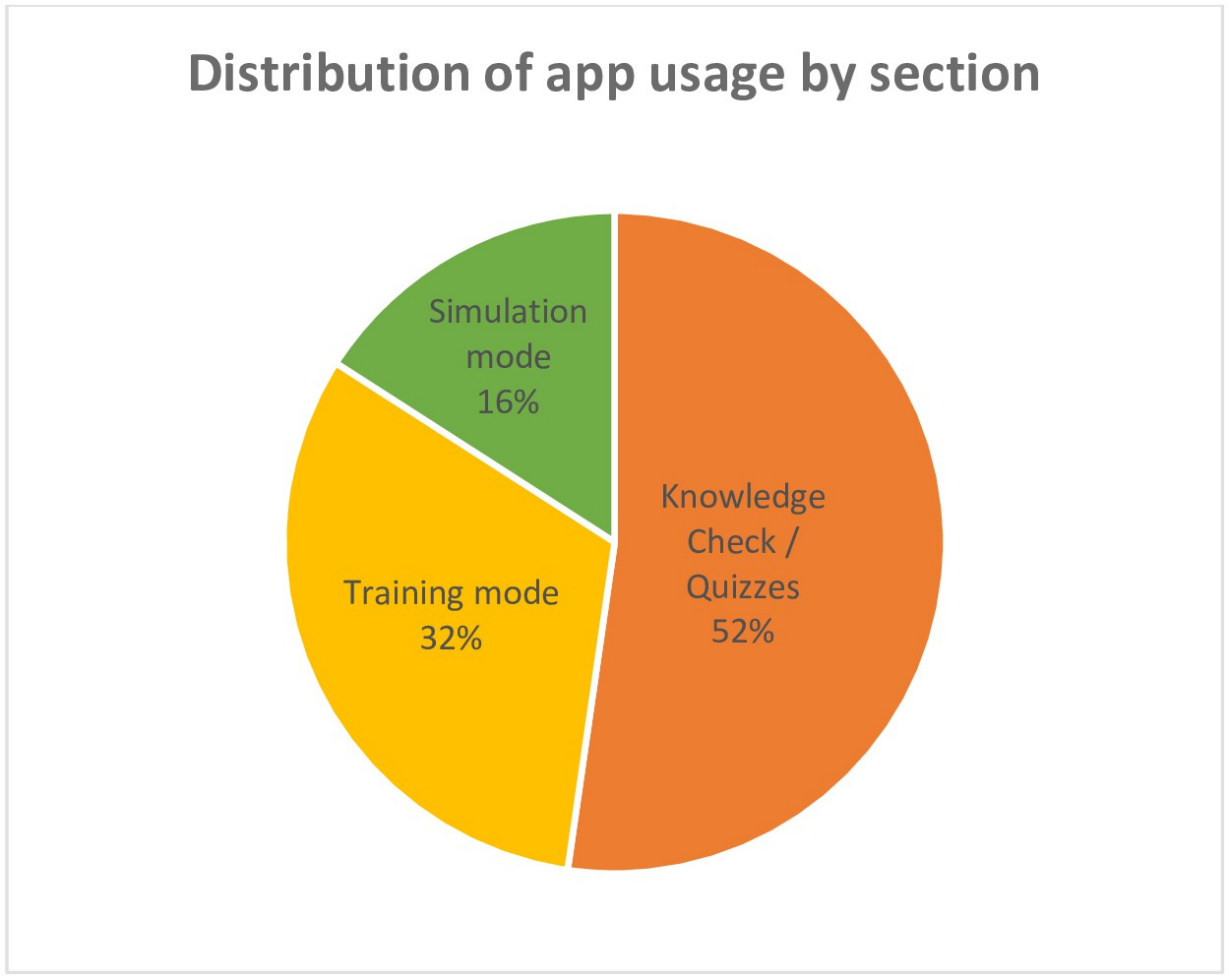
Proportion of app usage by app feature.

Of 17 trained users in the intervention group, six individuals (four of whom completed the six-month evaluation) used HBB Prompt with their own unique login. App usage varied across participants who used the app, and frequency of use increased three months after initial HBB training (Fig 5A and 5B). One user represented nearly half of the total logins, 17 out of 31. Of note, users continued to use the HBB Prompt after the intervention period, including users who had not previously logged in before. The most frequently used feature of the app was Quizzes/Knowledge check, followed by Training mode and Simulation mode (Fig 6). No users accessed the Scoreboard/Dashboard feature.

Using simple linear regression to examine the relationship between app usage and HBB skills and knowledge revealed no significant findings. No relationship was found between increased app usage and the OSCE score at six months, or the change in OSCE score immediately after training compared to six months after training. Similarly, there was no relationship between increased app usage or any app usage and knowledge check score at six months, or the change in knowledge check score immediately after training compared to six months after training. Simple linear regression to evaluate the influence of years of experience or deliveries per month on the OSCE score at six months, or change in OSCE score did not reveal any significant findings in both intervention and control groups.

Both intervention and control sites were asked to track their practice patterns using a logbook. In the intervention site, the logbook entries did not match the time stamps of app usage for nearly all the entries. Logbook data was requested but the logbook was not used by participants at the control site, although these healthcare workers did say they practised. However, because it was not possible to ascertain which users practised and the frequency of practice, it was not possible to conduct analysis on the influence of practice on OSCE performance in the control group.

### Focus group discussion findings

In the FGD at the control site, themes emerged regarding the importance of HBB training, the lack of continuing practice, disagreement between healthcare providers regarding neonatal resuscitation steps, and potential solutions to improve practice (Table 6). The discussion primarily focused on the lack of continuing practice and reasons that were given included not being required to be at a resuscitation, workload demands, and concerns of what colleagues would think of their skills if they were inadequate. Potential solutions included having a dedicated partner and reserved time for practice.

**Table 6:** Exit focus group discussion themes and sub-themes at control vs. intervention hospitals.

Themes	Sub-themes	Selected quotes
**Control**		
Importance of HBB Training		“We found it a satisfying experience, we get less or no asphyxiated babies with such problems.” “HBB is so, so helpful. If you use it, you save the baby.”
Lack of continuing practice	Not directly at resuscitations	“Some of us outside the department (maternity) do not come to practice with the manikin.”
	Workload	“you find that you cannot spare that time to come and practice if you know you are not going to be examined”
	Concern for what colleagues will think	“Sometimes the colleague feels shy to come (for practice) because if for example I resuscitated the baby and I didn’t succeed”
Disagreement between healthcare providers on steps of neonatal resuscitation		“But guess what, you call this medical officer, you want to do this, and they want to do that- the old resuscitation of babies. So, you find that you are clashing.”
Potential solutions to practice more	Have a dedicated partner	“I’m thinking of another solution for us the midwives who don’t actively work in this department, if we could pair up with a colleague here, it would work out as well”
	Have a dedicated time	“…before this shift goes [we] should come and we practise and spare something like 20 minutes then practise”
**Intervention**		
Allowing for consistency among providers		“we see resuscitation of the baby keeps on changing, and this is the current one that we are all practicing.” “almost all the team in the hospital are updated on the new guidelines.”
Usefulness as a clinical reminder		“Because you go into it, remind yourself of what you learnt, where you failed, it would give you the corrections” “This application has everything…It shows you do ABCD, up to the end.” “what I liked most were the videos because they were showing you the exact procedures. Where you went wrong, you would go back, refer to those videos, and correct yourself”
Importance of feedback		“And the beauty of that tablet is that, as you go through the questions, it gives you the scores you have gotten, and corrects you where you have gone wrong.”
Reasons app was not used	Password issues	“some of us forgot the passwords and it was hard to retrieve the passwords”
	Quizzes favored over simulation	“you would find when you are with a colleague down at the ward, you become busy a bit. So, you find yourself taking the app to your place where you are alone. So, it becomes easier to do the quizzes than this one which needs a colleague”
	Difficulty in finding partner	“Because you are in different shifts, you can’t find that you are together at the same time.”
	Challenge of only having one tablet to share	“If a midwife on the ward takes it, me who is here, I can’t access it.” “The other midwives who are not in maternity could not access it. So, once they have practised and they are done, the other people may not know that too unless they are informed that it is idle there and then they go and practise”
	Time constraints	“You find that even when there are three midwives on duty, they are not able to do all that.”
Improvement	Allowing users to access the app on their phones	“I have a request, can that app be modified that every one of us who wants, it can be given to her on her phone?”
	Sending reminders	“It was also very important for you to send us reminders …It will also remind us who have not gone there to practise”
	Visual display and/or reminders of other healthcare workers progress	“Like you show us- using our numbers of course, that “206, thanks for the practice, 201, you are reminded to practise”

At the intervention site, themes emerged regarding the utility of the app including the value of keeping consistency in practice to providers, the usefulness of the app as a clinical reminder, and the value of using the app to get feedback (Table 6). The participants stated that they had not used Simulation mode for a wide variety of reasons including the difficulty of finding partners who had time, workload demands, and only having the app available on one device in the hospital. They did say, however, that the videos and quizzes were used frequently as a clinical reminder. For improvement, several participants discussed the importance of receiving reminders. Feedback collected from anonymous surveys regarding HBB Prompt was consistent with findings from the FGD (Table 7).

**Table 7:** Feedback from intervention group regarding HBB Prompt.

Feature	Strongly Agree	Agree	Neutral	Disagree	Strongly Disagree
The voices were helpful	7	3			
Simulation scenarios were helpful	8	2			
The knowledge check questions were helpful	7	3			
The checklists within the app were helpful	7	2	1		
The dashboard within the app showing usage statistics were helpful	5	3	2		
Navigating within the app was easy		7	2	1	
The app was easy to use overall	1	7	2		
The app met my expectations	3	5	2		
The app functioned very well	6	3	1		

## Discussion

Over a six-month period, there was less skills decay in HCWs who had access to HBB Prompt, which had features that were developed to facilitate simulation-based practice of HBB skills. Our study results are in keeping with Umoren et al.’s [34] study on eHBB, which demonstrated the superior effect of supporting HBB training with VR technology compared to videos alone (passive reinforcement) or standard of care (access to HBB algorithm and teaching materials). In our study, the Simulation—Alone mode was used for the greatest amount of time, compared to other app features. This is similar to how eHBB is used as that program only allows for single user simulation. More broadly, these findings suggest that improving skills retention can be achieved with technology that supports simulation, an educational method that has gained increasing importance in a wide variety of pediatric and adult resuscitation education programs, including HBB [4, 35].

Overall, there was low frequency of practice captured by the app, with the highest app use during the latter part of the intervention period, potentially because participants knew there was to be an upcoming evaluation, or they recognized that knowledge and/or skills decay occurred and responded by refreshing skills and knowledge. Unfortunately, users in both sites did not adequately record their practice sessions using the logbook that was provided, and so practice trends could not be compared reliably between sites. Practice frequency may not have been well captured if users did not use the app as intended. Use of new technologies such as manikins that can capture practice frequency such as “NeoNatalie Live” (Laerdal Global Health) may mitigate this lack of objective data in the future [34]. Even though usage of HBB Prompt was low, it may be possible that horizontal transfer of knowledge occurred, or group practice occurred that was not captured by the way users accessed the app.

While both sites had decreasing rates of infants with asphyxia after HBB training, the decrease was only statistically significant in the intervention site. Additionally, while there was an upward trend of fresh stillbirth rates in the control site, there was a downward trend in the intervention site that appeared to be sustained six months after HBB training. Although these findings are encouraging, our study was not powered to discern whether these clinical trends were an effect of having access to the app or if they were due to other factors that were not captured in our dataset.

Lost to follow-up was significant in both groups, which further undermined the small number of participants in this pilot project. Although it is common for HCWs to be moved around from one health facility to another within regions in Uganda, the leaves of absence were not expected. It would have been difficult and inappropriate to exclude participants at the outset due to upcoming leaves. Another limitation regarding the healthcare workers in this study is that we could not capture informal methods of training prior to the study. Our demographic data specifically asked about formal course completion, and given that HBB has been in Uganda since 2010, some healthcare workers were exposed to HBB methodology prior to taking the formal course. The Ugandan Ministry of Health has also led initiatives to build capacity with HBB and provide facility-based mentorship to newborn providers. Of note, older HBB 1^st^ Edition posters were accessible in delivery rooms at both hospitals prior the start of this study. Data collection was affected by the COVID-19 pandemic and although the study protocol was set for 12 months after HBB training, issues with funding and the pandemic resulted in early termination of the study.

Similar to findings of other resuscitation training programs, knowledge retention does not translate to skills retention [10]. Therefore, ongoing efforts to improve sustainability of training need to focus on how to maintain resuscitation skills. Although Phase 1 [32] of this study attempted to address the issue of sustainability by focusing on UCD of HBB Prompt to suit end-user needs for acquisition and maintenance of skills of knowledge, the low frequency of usage of the app appeared to be mostly influenced by daily realities and limitations due to structural factors and daily workflow. While some of these issues were raised during the design phase, they were deemed out of scope of the development of HBB Prompt. The results of Phase 2 of this study suggest that more attention needs to be paid to address barriers in daily realities of HBB providers, such as workload, with the goal of integrating strategies for skills maintenance into daily workflow. Adding an extra tool like HBB Prompt that is not seamlessly integrated into the regular routine of HCWs may have been a reason for low app usage. Better mapping of the local HCW division of labour and workflow may have helped improve the ability to facilitate practice of HBB skills. For example, understanding the rotational nature of health workers going from labour and delivery to the postpartum and newborn wards and consideration of using natural transition points may improve uptake of HBB practice.

While health system challenges may be complex and difficult to address, employing UCD to understand the impacts of these challenges at the end-user level can still be advantageous in improving HBB Prompt or other solutions that increase sustainability of HBB. The scope of UCD can be expanded beyond just understanding how users will use a tool itself to more broadly understand how the tool will be integrated within the existing environment. Through an iterative process of UCD, systems-based gaps can be identified, and solutions tested. Use of UCD is still a worthwhile undertaking as it tailors interventions to the needs of end-users, and can improve efficiency and effectiveness of solutions.

Examining various aspects of usage can help better understand trends. For example, frequency of access versus duration of access may differ depending on the nature of how users interacted with the app. In this study, the total and average time spent in Simulation mode was higher (total 104 minutes, average 5.8 minutes) than in Training mode (total 50 minutes, average 1.8 minutes). Within Simulation mode, only Simulation—Alone was utilized and not the Simulation—Group function. This may have been because users preferred to do their own simulations, or they may have had difficulty in finding peers for group practice, as they indicated in the FGDs. Although simulation is a methodology used in HBB training, using the app to practise is different than going through a peer or trainer-led simulation. Although one intention of creating Simulation—Alone mode was to facilitate individual simulation-based practice, users may not have spent adequate time training to be able to use this feature confidently on their own. More time spent with the target end users in the user-centred design cycle may have been able to uncover any additional app functions and features to improve usage. In terms of frequency, quizzes were the most accessed part of the app, followed by the training videos and Simulation mode. The self-study nature of quizzes, ease of use and instant gratification of getting answers marked as correct may have influenced the higher frequency of access to quizzes. Tracking app usage through built-in analytics enabled better understanding of user behaviour. However, one significant limitation to understanding app usage and its impact on skills decay was that one user represented nearly half of all logins. This likely affected the ability to determine any meaningful relationships at the group level when evaluating the link between app usage and intervention outcomes such as skills retention. Despite this limitation, there was a distinct difference in less skills decay in the intervention group, which may be secondary to potential unmeasured effects of having access to the app.

Having the app deployed on one tablet for the ward rather than on personal devices made it more difficult to coordinate access. On the other hand, it would have been challenging to demand specific technical requirements for personal devices or to offer internet credit in order to have their app analytic data uploaded. During Phase 1, participants had more time exposed to the app with the guidance of the study team. More streamlined tech support may have been helpful as access and login were cited as important initial barriers to using the app. During the FGD participants did not identify significant difficulties in using the app; however, they may not have been forthcoming with their true thoughts since the facilitators of the FGD were the study investigators. Similarly, the positive review of the app and its features as captured by the post-intervention survey was not congruent with the lack of app usage. This highlights the importance of ongoing and dedicated user-centred feedback to truly capture the nuances of how technology can be integrated into user’s daily activities, workflow and ultimately how to continue to adapt technology in a way that truly benefits users and targeted outcomes such as skills retention.

Reminders and strategies to prompt LDHF practice have been shown to be effective in other settings [17–20, 36]. The Scoreboard/Dashboard feature was created to help hold users accountable for their own practice habits and those around them; however, this feature was not utilized by the users at all. One potential explanation is that this feature is a passive way to motivate users to practise their skills. Employing strategies that rely less on users to login to the app to practise in the first place may be useful. For example, perhaps the dashboard could have been displayed at a central location, and linked to the tablet so that it would have been prominently displayed to trigger participants to turn on the app and practise. Specific targeted reminders that change with participant behaviour may be helpful as an improvement. For instance, if participant A has not practised in a month versus participant B has practised once a day, A’s reminder would highlight the lack of practise, and encourage them to start practising and B’s reminder would be more positive reinforcement.

Skills retention remains a significant challenge for sustainability of impact of initial HBB training. Refresher trainings and increased supervision are strategies that have been shown to improve retention [16, 20, 37]; however, these methods are resource intensive. Technology based solutions that are integrated into daily routines of users, such as mobile apps like HBB Prompt, enhanced manikins like “NeoNatalie Live” [34, 36] or VR technology like eHBB [34] may provide feasible solutions that are more easily scalable. Without specific integration into daily workflow, there was a trend towards improved retention with use of HBB Prompt, similar to eHBB VR. With ongoing careful design and integration into the routines of health workers at the frontlines, there is potential for technologies to exert an even larger impact [38].

## Conclusions

There was less neonatal resuscitation skills decay in HCWs with access to HBB Prompt, despite low usage. Further studies on use of mobile apps to improve skills retention should focus on integration of the app into the daily routines of end-users to maximize the app’s utility.

## Supporting information

S1 Text Focus group discussion guide for control site at 6 months. (DOCX)

S2 Text Focus group discussion guide for intervention site at 6 months. (DOCX)

S3 Text HBB Prompt study protocol version 2018–02. (PDF)

S1 Data HBB Prompt phase 2 data. (XLSX)

S1 Checklist CONSORT checklist. (DOC)
