# Supplementary material for: Utilization of a novel mobile application, “HBB Prompt”, to reduce Helping Babies Breathe skills decay
Source: PLOS Glob Public Health. 2023 May 8;3(5):e0000705. doi: 10.1371/journal.pgph.0000705 (PMC10166562; doi:10.1371/journal.pgph.0000705)
Supplement: S1 Text — (DOCX) [file pgph.0000705.s001.docx]

**Control Group - HBB Prompt Phase 2 – 6 month FGD Guide**

**Introduction focus group discussion: (15 minutes)**

Prep: voice recorder on table and backup recording on IV pole mounted tablet

Setup: participants seated in roundtable

Audio recording: yes

|  | - Introduction to FGD – Ask participants to identify themselves at the beginning with their participant ID (given to them on a masking tape they will wear) and to please do this every time a recording is started |
| --- | --- |
|  | - - Facilitators to provide example of how it works: e.g. “701: I think bagging is very hard” “702: I disagree, I think keeping a baby warm is harder” |

**Focus group discussion (~ 1.5 hour)**

1. **Broad/open ended questions to start:**
   1. How was it?
   2. Did the training meet your needs?
   3. In what way did the training help?
   4. In what way was the training lacking?
   5. How was it to practise on shift?

* Can ask about before/after

1. **Questions about HBB**
   1. What are challenges that make it hard to retain/maintain HBB skills
   2. What would be helpful to you to assist with retaining knowledge and skills?
   3. Tell us about your workload as a provider in your unit and how it relates to your desire and ability to maintain HBB skills.

**15 Minute Break**

**Wrap-up**

Prep: voice recorder

Thank all participants and have them reflect on what they learned today and what they feel can be further addressed
