# Supplementary material for: Utilization of a novel mobile application, “HBB Prompt”, to reduce Helping Babies Breathe skills decay
Source: PLOS Glob Public Health. 2023 May 8;3(5):e0000705. doi: 10.1371/journal.pgph.0000705 (PMC10166562; doi:10.1371/journal.pgph.0000705)
Supplement: S2 Text — (DOCX) [file pgph.0000705.s002.docx]

**Intervention Site - HBB Prompt Phase 2 – 6 month FGD Guide**

**Introduction focus group discussion: (15 minutes)**

Prep: voice recorder on table and backup recording on IV pole mounted tablet

Setup: participants seated in roundtable

Audio recording: yes

|  | - Introduction to FGD – Ask participants to identify themselves at the beginning with their participant ID (given to them on a masking tape they will wear) and to please do this every time a recording is started |
| --- | --- |
|  | - - Facilitators to provide example of how it works: e.g. “701: I think bagging is very hard” “702: I disagree, I think keeping a baby warm is harder” |

**Intervention - Focus group discussion (~ 1 hour)**

1. **Broad/open ended questions to start:**
   1. How was it?
   2. Did the app meet your needs?
   3. In what way did the app help?
   4. In what way was the app lacking?
   5. How was it to practise on shift
2. **Specific Questions about the app**
   1. In what situations was it useful?
   2. What did you think of the content?
   3. What did you think of the interface?
   4. How easy was it to navigate within the app?
   5. How did it function overall?
   6. Were you able to use it with practised simulation?
   7. Any suggestions for further improvement?
3. **Questions about HBB**
   1. What are challenges that make it hard to retain/maintain HBB skills
   2. What would be helpful to you to assist with retaining knowledge and skills?
   3. Tell us about your workload as a provider in your unit and how it relates to your desire and ability to maintain HBB skills.
4. **Questions based on user questionnaire**
   1. What feature of the app was most helpful

**15 Minute Break**

**Activity: Using HBB Prompt (~ 1.5 hours)**

Prep:

- 2 tables each set up with a neonatalie kit. Divide group into two.
- voice recorder for each station recording – assign each one to one facilitator to manage
- voice recorder on table and backup recording on IV pole mounted tablet for group discussion

Setup: participants work in pairs to utilize HBB prompt with the NeoNatalie^TM^

Audio recording: yes

Explain activity:

- We would like to see how you use HBB Prompt with a partner in doing the simulation practice. You will have **15 minutes** to do this with a partner, and after each pair has had a chance to use it, we will come together to discuss your ideas as a group.

**Wrap-up**

Prep: voice recorder

Thank all participants and have them reflect on what they learned today and what they feel can be further addressed
