# Supplementary material for: Utilization of a novel mobile application, “HBB Prompt”, to reduce Helping Babies Breathe skills decay
Source: PLOS Glob Public Health. 2023 May 8;3(5):e0000705. doi: 10.1371/journal.pgph.0000705 (PMC10166562; doi:10.1371/journal.pgph.0000705)
Supplement: S3 Text — (PDF) [file pgph.0000705.s003.pdf]

HBB Prompt: Development of a mobile application through user-centered design to improve Helping Babies Breathe skills retention

Principal Investigators: **Dr. Santorino Data, MBChB, MMed Pediatrics and Child Health. Mbarara University of Science and Technology**

Dr. Natalie Hoi-Man Chan, Research Collaborator, The Hospital for Sick Children

Co-Investigators

Hasan Merali, Associate Staff Physician, Pediatric Emergency Medicine, The Hospital for Sick Children
Email: hasan.merali@sickkids.ca

Niraj Mistry, Staff Physician, Pediatrics, The Hospital for Sick Children
Email: niraj.mistry@mail.utoronto.ca

Douglas Campbell, Staff Physician, Neonatology, The Hospital for Sick Children and St. Michael's Hospital Email: Campbelld@smh.ca

Ryan Kealey, PhD Candidate, Interactive Media Lab, University of Toronto
Email: ryan.kealey@gmail.com

Shaun Morris, Clinician-Scientist, Infectious Diseases, The Hospital for Sick Children Email: shaun.morris@sickkids.ca

HBB Prompt: Development of a mobile app through user-centred design to improve Helping Babies Breathe skills retention

Funding

Grand Challenges Canada Stars in Global Health R-ST-POC-1707-07454
Program coordinator: Anne-Isabel Cameron

Background and rationale

Birth asphyxia causes up to 1.8 million newborn deaths and stillbirths every year. This global tragedy can be prevented by effectively Helping Babies Breathe at the time of birth. Helping Babies Breathe (HBB) (Appendix 1) is a newborn stabilization course that has been shown to reduce early neonatal mortality by up to 47% in Tanzania.(1) Significant reduction in intrapartum still births and first day mortality have also been demonstrated in other low and middle-income countries. (2, 3) The knowledge and skills acquired in this course, however, have been shown to rapidly deteriorate over time, with skills deteriorating more than knowledge. (4) The largest systematic review on this topic suggests that although not commonly included in resuscitation programs, refresher training is likely necessary to improve the impact on neonatal mortality.(5) Mduma et al have demonstrated that frequent brief on-site simulation with the HBB program can have a sustained impact on mortality.(6) Furthermore, regular, low intensity, manikin-based skills training with peers has been shown to allow workers to maintain bag mask ventilation skills at one year. (7) We propose to improve sustainability of HBB's impact by increasing skills retention with an innovative mobile application called HBB Prompt. If successful, our application provides a novel, low-cost method for providers to receive regular simulation training with peers.

HBB Prompt will be an interactive tool that guides frontline providers through the steps needed to save newborn babies at birth. HBB Prompt will facilitate individual and group training in health facilities using the Low Dose High Frequency model (LDHF) for resuscitation skills retention. Our group has successfully developed and tested a related application for the Neonatal Resuscitation Program (NRP), the newborn stabilization algorithm used in North America.(8) We will use lessons learned from our previous work to robustly develop HBB Prompt by integrating human factors and user-centered design approaches. We will engage end-users and HBB Master Trainers to iteratively collect feedback to develop HBB Prompt for both individual and small group resuscitation practice. Our iterative approach will mitigate the common scenario of mobile health (mHealth) solutions unable to achieve sustained success at scale due to lack of comprehensive input from frontline users. We will pilot the app at a single centre and compare it to a control site for HBB skills retention.

Hypothesis: Applying a user-centered design approach of collecting iterative qualitative data from end-users regarding learning needs and facilitators and barriers to HBB skills maintenance will enable creation of a mobile app that will improve skills retention in an intervention group versus a control group.

Objectives

1. To develop a refined HBB Prompt app through user-centered design involving input from frontline birth attendants in Uganda.
2. To train a minimum of twenty health workers from two district hospitals in Southwestern Ugandan in HBB 2.0 and Essential Care for Every Baby within 6 months of the start of the project.
3. To determine the impact of the HBB Prompt app on HBB skills retention of providers in the intervention site compared with providers in the control site (higher average OSCE B test scores) 12 months after training.

Objective 1: To develop a refined HBB Prompt app through user-centered design involving input from frontline birth attendants in Uganda.

Phase 1a

We will collect observational data on the process of HBB training and HBB skills in action to determine barriers and habits of formation that could be redirected or improved. This processes and skills habits may not be apparent to end-users. This will be conducted by review of audio-visual footage of HBB training sessions. Video recordings will be obtained using roof mounted video equipment from the Mbarara University Simulation center after full participant consent.

We will then solicit qualitative feedback regarding the perceived facilitators and barriers to learning and maintaining HBB knowledge and skills through individual interviews and separate focus groups organized by provider level of experience to allow for exploration of different needs for skills acquisition and skills maintenance. We aim to recruit 4-6 participants per group to reach saturation of themes.

Focus group composition will be:

1) HBB Master trainers and facilitators,
2) Target end users of the HBB program such as previously trained HBB providers and
3) Pre-service trainees (total of 3 groups).

The anticipated provider types within each group would include the following:

- Nurses and midwives
- Medical officers

- Consultant physicians (pediatricians)
- Medical and nursing students typically in their clinical rotations.

Interview and focus group questions will address:

- Personal experiences regarding successes and challenges in stabilization of the newborn
- Barriers encountered and perceived needs to enable optimal maintenance of skills
- Personal experiences regarding successes and challenges of the actual HBB training sessions. Discussion about which parts enhanced their learning (e.g. simulation, graphics, anecdotes from others, etc) and which parts made it difficult to learn skills
- How they would design a solution to aid them (participatory design) to overcome barriers and/or to enhance their ability to use and maintain HBB knowledge and skills
- Whether they have participated in any refresher courses and what they thought of the utility of refresher training

Phase 1b: Development of HBB Prompt app through user-centred design

1. With the assistance of the project team's knowledge and previous experience with neonatal resuscitation and HBB, a team of engineers from Mbarara University of Science and Technology (MUST) will work together with the investigator team to create a prototype app, HBB Prompt v0. The app will be designed on the Android platform. The Android platform was chosen for a number of reasons including a high usage of Android devices in Uganda, being open source and accessible on inexpensive devices, which will make the app more amenable for scale-up.
2. With input from the team's specialist in human factors and user-centred design, data gathered from Phase 1a will be integrated into HBB Prompt v0 to create HBB Prompt v1.
3. Phase 1b.1 to Phase 1b.3 will be an iterative process of feedback and adaptation of the app where usability and feasibility testing of the prototype app will be conducted to determine the optimal and desired features prior to pilot testing in Phase 2a.
   a. Providers from the same groups in Phase 1a will participate in three sessions (Phases 1b.1 – 1b.3) where they will have time to explore the app and then use the app in simulated neonatal resuscitation training, after which they will provide feedback in focus group discussions and in short interviews. This process will allow the users to better understand how the app may work in real time to provide targeted suggestions to further improve the app. Both focus group discussions and interviews will be voice recorded transcribed and analyzed to inform further app development.
   b. Focus group discussion questions will address:
      i. content, interface, navigation, functionality, customizability, usability
      ii. where participants see the use of the app taking place

   iii. how can the app be further modified or improved to achieve their desired functionality or fill the gaps that are identified with HBB learning
c. The goal is to have:
   i. 5 participants per group type (3 groups) = 5 x 3 = 15
   ii. Interviews (15-30min/) for experience level (3 x 3)

Objective 2: To train a minimum of twenty health workers from two district hospitals in Southwestern Ugandan in HBB 2.0 and Essential Care for Every Baby (ECEB) within 6 months of the start of the project.

Phase 2a

We will train a minimum of 20 frontline health providers (10 per hospital) from two district hospitals in southwestern Ugandan in HBB 2.0 and ECEB. HBB and ECEB training will be conducted by national trainers in the ratio of 1 trainer to 6 providers. Providers will undergo ECEB training in addition to HBB as these training programs are recommended by the Uganda Ministry of Health to be offered together.
HBB 2.0 training takes place over one day and ECEB training over two days. Participants will be invited from both hospitals to any one episode of training to minimize the number of frontline birth attendants in trainings at any single time.
One hospital will be randomly selected and its providers will have access to the most updated version of HBB Prompt (beta) at the end of the trainings (Intervention arm). The control group will not have exposure to the HBB Prompt app post training.

Participants in both centers will be asked to achieve a minimum practice target of once per day. Our recommended frequency to use the app will be once per shift (i.e. run through 1 simulation per person as a pair). An HBB article from earlier this year showed the greatest improvement when health workers practiced at the beginning of every shift. As it may be difficult to ensure that it is done at the beginning of shift, we will leave the timing of once a shift practice at the discretion of providers. Whenever a new team comes on, they will be recommended to ask the previous team if they did a simulation practice. If not, then they will remind the outgoing team to complete it. These recommendations on frequency of practice will be offered at both the intervention and control sites. All sites will receive resuscitation practice mannequins and a resuscitation practice table.

We will present to the Health Facility team the results of their HBB simulation practice frequency as captured by the HBB Prompt app analytics in the intervention arm. This app analytics data on frequency of practice will be presented to the health facility every two months during the one year study period. The app will be designed to generate both individual and group (facility) practice frequency. Both intervention and control arms will have a standard log-book to record frequency of practice. The accuracy of the log-book will be compared to the data retrieved from app analytics in the intervention arm.

Objective 3: To determine the impact of the HBB Prompt app on HBB skills retention of providers in the intervention site compared with providers in the control site.

1. Primary outcome: comparison of OSCE B scores in intervention (HBB Prompt) vs. control group. (17 out of 23 constitutes a pass score)
    a. At 0 months, (immediately before and after training)
    b. At 3 months
    c. At 6 months (midline)
    d. And at 12 months (endline)
2. Measures during the 12-month period of intervention in HBB Prompt site and control site at two unannounced visits (4 months, 9 months):
    i. HBB 2.0 Knowledge check (18 questions)
    ii. Bag and mask ventilation skills check (score out of 14)
    iii. OSCE A (score out of 12, 9 out of 12 constitutes pass score)
    iv. In addition to the OSCE skills checklist as scored by raters, we will use the Augmented Infant Resuscitator (AIR) to objectively measure quality of newborn resuscitation during every ventilation epoch. AIR records time stamped data on ventilation quality, such as presence of air leak or obstruction and ventilation rate (www.air-device.com).

    b. Data on potential confounders will be collected for each site (volume of deliveries, workload in labor and delivery, burnout, motivation, baseline supervision, experience, previous training, provider level, number of patients/areas each provider is responsible for, length of time provider has been in practice, availability of equipment, availability of an HBB algorithm posted at the bedside.)
    c. Other clinical outcomes will be collected from the Ministry of Health Birth Register: For each patient: date of birth, time of birth, birth weight, death before discharge, admission to nursery, 1 and 5 minute Apgar scores, resuscitation status at birth and resuscitation outcome.
    d. Reports generated and presented to each site every 2 months: frequency of practice, number of deliveries, number of infants requiring bagging, fresh stillbirths, neonatal mortality in hospital
3. App analytics
    a. Pattern of usage
        i. Which components of the app are being used?
            - training mode, simulation mode, help videos, quizzes
    b. Frequency (login, page use), purpose device ID
        i. Is low dose high frequency happening? – Frequent, short duration access?
    c. Self practice vs. peer or group practice
    d. Trends for scores for simulations and quizzes

4. We will conduct exit focus group discussions and interviews at both intervention and control sites:
    a. For Intervention site providers:
        i. Facilitators and barriers to implementing and maintaining HBB skills
        ii. Did HBB Prompt help?
        iii. Did HBB Prompt meet end-user needs
        iv. Any suggestions for further improvement
        v. Feasibility and sustainability of scale-up
        vi. HBB Prompt specifics: content, interface, navigation, functionality, customizability, usability
    b. For Control site providers:
        i. Facilitators and barriers to implementing and maintaining HBB skills
        ii. Discussion about ideas to facilitate skills maintenance
    c. For both sites:
        i. Perception of workload in labor and delivery, burnout, motivation
        ii. Interviews only: description of baseline supervision, number of patients and areas each provider is responsible for, availability of equipment for practice and for clinical care (e.g. bag-mask ventilator, HBB algorithm)

Randomization

A random sequence generator will be used to determine a random number just prior to the start of phase 2. If the random number is odd, then Itojo will be the intervention site. If the number is even, then Kitagata will be the intervention site.

Study setting

Southwestern Ugandan (Itojo Hospital, Ntungamo District; Kitagata Hospital, Sheema District). Each study site will have HBB practice corners established and equipped with both resuscitation mannequins and resuscitation bag and mask devices.

Study duration

18 months in total, 4 months for phase 1 and 12 months for phase 2 and 2 months for data analysis.

Participant eligibility criteria and enrolment

Study Population Eligibility: We will conduct the study in an area where HBB is recommended as the training program for newborn resuscitation

Inclusion Criteria: Frontline birth attendants involved in the delivery or care of babies in the maternity ward, theater or pediatric wards with a possibility of involvement in newborn resuscitation.

Exclusion Criteria: Health workers providing care in other wards other than maternity and pediatrics.

Enrolment: Using community entry processes, the medical superintendents of each hospital will be approached and requested to allow the study to be conducted in the Hospital. Once consent is obtained from the superintendent, individual frontline birth attendants will be approached and consented to be part of the study before training sessions are conducted.

Data Analysis

Phase 1

Audio recordings from focus groups and interviews will be transcribed. Transcripts will be reviewed for themes using a grounded theory approach. Data analyses will take place after each iteration of phase 1b to incorporate feedback to improve and adapt each version of the HBB Prompt prototype. At least two investigators will independently read all transcripts at least twice to obtain an overall understanding, identify data codes and ensure that all comments are carefully considered and included. Categories within the themes will then be identified, and any relationships among themes determined. As data are entered into the analyses, categories will continue to be generated, until there is no new data that cannot be categorized under existing codes. When this occurs, it is likely that a range of functions and features of an ideal mobile app as a learning tool for HBB will have been categorized. Further, we will also likely have categorized an assortment of barriers and facilitators to skills maintenance and implementation of HBB Prompt. A similar analytic approach has previously been successfully used in the development of an osteoporosis clinical decision support tool (10).

Phase 2

Comparison of intervention versus control for the following outcomes will be analyzed at each time point using the Mann-Whitney U test:

- Primary outcome: OSCE B score at 12 months
- Secondary outcomes:
  - OSCE B scores at 0, 3, and 6 months
  - At two unannounced site visits (months 4 and 9)
    - Knowledge check score
    - BMV skills check score
    - AIR data
      - Percentage of time with correct ventilation rate
      - Percentage of time with air leak during ventilation
      - Percentage of time with obstructed airway during ventilation
      - Time to effective ventilation
    - OSCE A score

Additionally, comparison of OSCE B score after training at 0 months and at 12 months within each arm using the Wilcoxon-signed rank test

	0 months	3 months	4 months	6 months	9 months	12 months
OSCE B	Pre/post HBB training	x		x		x
OSCE A			x		x	
Knowledge check			x		x	
Bag-mask skills			x		x	
AIR			x		x	

Descriptive statistics compared between intervention and control for:

- Demographic data and potential confounders (volume of deliveries, workload in labor and delivery, burnout, motivation, baseline supervision, experience, previous training, provider level, number of patients/areas each provider is responsible for, length of time provider has been in practice, availability of equipment, availability of an HBB algorithm posted at the bedside)
- Other clinical outcomes: death before discharge, admissions to the nursery, number of deliveries, number of infants requiring bagging, fresh stillbirths, neonatal mortality prior to discharge
- Frequency of practice, self vs. group practice, skills practiced

Qualitative data from focus groups and interviews:

- Similar to in phase 1, audio recordings will be transcribed and reviewed using a grounded theory approach. Coded themes and categories will be compared descriptively between participants in the intervention versus the control groups.

Budget Summary

Budget category	Total CAD
Remuneration - funding recipient's employees	35,441
Fees - Subcontractors	19,642
Reimbursable Travel Costs	9,960
Reimbursable Goods and Supplies	9,304
Reimbursable Equipment Costs	2,640
Reimbursable Project Administration Costs	2,845
Sub-grants	20,000
**Total Project in CAD**	**99,831**

References

1. Msemo G1, Massawe A, Mmbando D, Rusibamayila N, Manji K, Kidanto HL, Mwizamuholya D, Ringia P, Ersdal HL, Perlman J. Newborn mortality and fresh stillbirth rates in Tanzania after helping babies breathe training. Pediatrics. 2013 Feb;131(2):e353-60. doi: 10.1542/peds.2012-1795. Epub 2013 Jan 21.
2. Kc A1, Wrammert J2, Clark RB3, Ewald U2, Vitrakoti R4, Chaudhary P4, Pun A2, Raaijmakers H2, Målqvist M2. Reducing Perinatal Mortality in Nepal Using Helping Babies Breathe. Pediatrics. 2016 Jun;137(6). pii: e20150117. doi: 10.1542/peds.2015-0117.
3. Goudar SS1, Somannavar MS, Clark R, Lockyer JM, Revankar AP, Fidler HM, Sloan NL, Niermeyer S, Keenan WJ, Singhal N. Stillbirth and newborn mortality in India after helping babies breathe training. Pediatrics. 2013 Feb;131(2):e344-52. doi: 10.1542/peds.2012-2112. Epub 2013 Jan 21.
4. Bang A, Patel A, Bellad R, Gisore P, Goudar SS, Esamai F, et al. Helping Babies Breathe (HBB) training: What happens to knowledge and skills over time? BMC Pregnancy Childbirth. BMC Pregnancy and Childbirth; 2016 Nov 21;:1–12.
5. Reisman J, Arlington L, Jensen L, Louis H, Suarez-Rebling D, Nelson BD. Newborn Resuscitation Training in Resource-Limited Settings: A Systematic Literature Review. Pediatrics. 2016 Aug;138(2). pii: e20154490. doi: 10.1542/peds.2015-4490. Epub 2016 Jul 7.
6. Mduma E, Ersdal H, Svensen E, Kidanto H, Auestad B, Perlman J. Frequent brief on-site simulation training and reduction in 24-h neonatal mortality—An educational intervention study. Resuscitation. 2015 Aug;93:1-7. doi: 10.1016/j.resuscitation.2015.04.019. Epub 2015 May 6.
7. Arabi AM, Ibrahim SA, Ahmed S2, MacGinnea F, Hawkes G, Dempsey E, Ryan CA. Skills retention in Sudanese village midwives 1 year following Helping Babies Breathe training. Arch Dis Child. 2016 May;101(5):439-42. doi: 10.1136/archdischild-2015-309190. Epub 2016 Jan 29.
8. Wyckoff MH, Aziz K, Escobedo MB. American heart association guidelines update for cardiopulmonary resuscitation and emergency cardiovascular care: Part 13: neonatal resuscitation. Circulation; 2015.
9. FDIs, I. (2009). 9241-210 (2009). Ergonomics of human system interaction-Part 210: Human-centered design for interactive systems (formerly known as 13407). International Organization for Standardization (ISO). Switzerland.
10. Krueger RA, Casey MA. Focus Groups: A practical guide for applied research. 4th ed. California: Sage Publications Inc., 2008.
